# Bibliometric analysis of “sarcopenia” in Web of Science

**DOI:** 10.1186/s43166-023-00194-w

**Published:** 2023-05-30

**Authors:** Tuba Tülay Koca

**Affiliations:** Faculty of Medicine, Department of Physical Medicine and Rehabilitation, Sütçü İmam University, Kahramanmaraş, Turkey

**Keywords:** Sarcopenia, Bibliometric analysis, Bibliometrics, Rheumatology, Rehabilitation

## Abstract

**Background:**

Sarcopenia is defined as age-related muscle wasting defined by the combination of appendicular muscle mass, muscle strength, and physical performance measures. Here, we made a bibliometric analysis of the publications published in the Web of Science (WoS) between 2018 and 2023 in terms of “sarcopenia.” In total, 12,461 articles were analyzed. The authors, publication year, title, publishing country/journal/institution, keywords, Web of Science categories, publishers, indexes, citation topics, and reports were reviewed for each article. All digital researches were done on January 03, 2023.

**Results:**

Among 12,461 articles, according to documents types, 8855 were research articles, 1793 were reviews, 1028 were meeting abstracts, 3963 were early accesses, 364 were letters, and others (proceeding paper, correction, book chapter, etc.). A total of 7446 was open accesses.

According to the number of papers and according to publication year, 3537 were in 2021 (highest), 3349 were in 2022, 3024 were in 2020, 2532 were in 2019, and 19 were in 2023. According to the top 5 Web of Science categories list, 2792 were in Geriatrics Gerontology, 1857 were in Nutrition Dietetics, 1575 were in Medicine General Internal, 983 were in Oncology, and 914 were in Endocrinology and Metabolism. Additionally, 243 papers were in Sport Sciences, 201 were in Rehabilitation, 196 were in Rheumatology, and 187 were in Clinical Neurology.

Top citation topics were Nutrition and Dietetics (6225), Musculoskeletal Disorders (803), Bone Disease (251), Urology & Nephrology-General (236), and Hepatitis (163) (respectively). The number of citing articles, between 2019 and 2023 years, was 39,534 (with self-citations) and 39,088 (without self-citations); times cited was 94,584 (with self-citations) and 69,585 (without self-citations).

**Conclusion:**

Our study highlights the characteristics of researches in sarcopenia and provides an objective insight into the importance of sarcopenia in elderly and patients with chronic rheumatic diseases.

## Background

Sarcopenia refers to loss of muscle strength and mass. Primary sarcopenia is the loss of muscle mass associated with advanced age. Secondary sarcopenia (disease-associated sarcopenia) focuses on muscle mass loss regardless of muscle function. These diseases include cancer, chronic obstructive pulmonary diseases, heart failure, and kidney failure. Secondary sarcopenia also includes treatment of the underlying disease. The elderly population is increasing around the world, and together with this, the quality of life and healthcare expenses of the elderly population have become the major subject of research and public health policies. Despite its clinical importance, sarcopenia has not been adequately diagnosed and controlled in routine practice [1].

The prevalence of sarcopenia varies about 6–22% in elderly individuals. Its prevalence varies depending on gender, age, pathological conditions, and diagnostic criteria. It is generally undiagnosed health problem. It is seen in older people in relation with age and can be affected by genetic and lifestyle factors. Physical activity and nutrition are the main ways to prevent sarcopenia. The diagnosis of sarcopenia is based on muscle mass measurements and functional tests that assess muscle strength or physical performance. The pathogenesis of sarcopenia is complex and multifactorial. Physical activity alone or in combination with protein supplementation has been shown to be effective in preventing disability and fragility in older people by increasing muscle mass and strength [1–3].

Although the relation of sarcopenia with endocrine disorders or malignancies is well known, sarcopenia is frequently seen in many autoimmune diseases (rheumatoid arthritis, spondyloarthropathies, systemic sclerosis, inflammatory bowel diseases, and autoimmune diabetes), especially due to chronic inflammation. In addition to chronic inflammation (high acute phase reactants), advanced age, high body fat, long disease duration, bone erosions, low hip bone mineral density, malnutrition, low protein intake, and joint damage are also closely associated with sarcopenia. When we look at the studies in the field of rheumatology, although the relationship between rheumatoid arthritis and sarcopenia is well defined, its relationship with other autoimmune diseases is not sufficient [4]. In addition, it is seen that the parameters evaluating sarcopenia differ in studies. Rarely, “sarcopenic obesity,” in which muscle loss is replaced by adipose tissue, is also ignored [5–7]. Hospital costs are also higher for sarcopenic patients than non-sarcopenic patients.

In the absence of pharmacological drugs in the treatment of the disease in sarcopenic patients, non-pharmacological measures appear as the only option. With the regulation of effective preventive treatment strategies, sarcopenia will be included in our routine clinical practice. Currently, there is a need to provide a better diagnosis of sarcopenia, diagnostic tools, precautions, and individual health care.

Bibliometry is a numerical analysis of the publications produced by individuals or institutions in a certain area, in a certain period, and in a certain region and the relations between these publications. Bibliometric methods have been used in the medical disciplines for more than 30 years. Web of Science (WoS) consists of multidisciplinary citation indexes belonging to Clarivate Analytics Company, covering scientific journals with high-impact factor worldwide.

Our aim is to analyze the currently published literature in the WoS on sarcopenia by bibliometric method. By this analysis, we aimed to provide an objective perspective to the documents in different regions and disciplines all around the world and to update the current literature data from the perspective of a rheumatologist. The study could be beneficial in determining sarcopenia research priorities and the importance of scientific research on this subject.

## Methods

### Data source

A systematic evaluation of the literature using the Web of Science database (https://www.webofscience.com/wos/woscc/basic-search) (WoS) was made between 2018 and 2023 years. The search term “sarcopenia” was used by putting the first row. In total, 12,461 articles were analyzed. The reasons for choosing the WoS database are the number of publications they include, especially in social and human sciences, their subject distribution, and geographical distribution. Additionally, it is possible to access the WoS database in all universities in our country.

### Data collection

The authors, publication year, title, documents types, publishing country/journal/institution, keywords, Web of Science categories, publishers, indexes, citation topics, and reports were reviewed for each article. Descriptive data of the analysis was conducted via Microsoft Excel 2010, and Web of Science database’s graphics were also used. All digital researches were done on January 03, 2023. All indexes, Emerging Sources Citation Index (ESCI), Science Citation Index-Expanded (SCI-E), and Social Science Citation Index (SSCI), were included in the study.

### Analysis and visualizations

Using Microsoft Excel 2010, the data in the tables were converted to absolute values (percentage and frequency). The visualizations from the WoS database were also utilized. Descriptive statistical methods were used mostly in this study. Graphs were also made by using IBM SPSS 21.0 program (SPSS Inc., Chicago, IL, USA). Citation reports are only available for 10,000 records or fewer in WoS database.

## Results

**Fig. 1:**
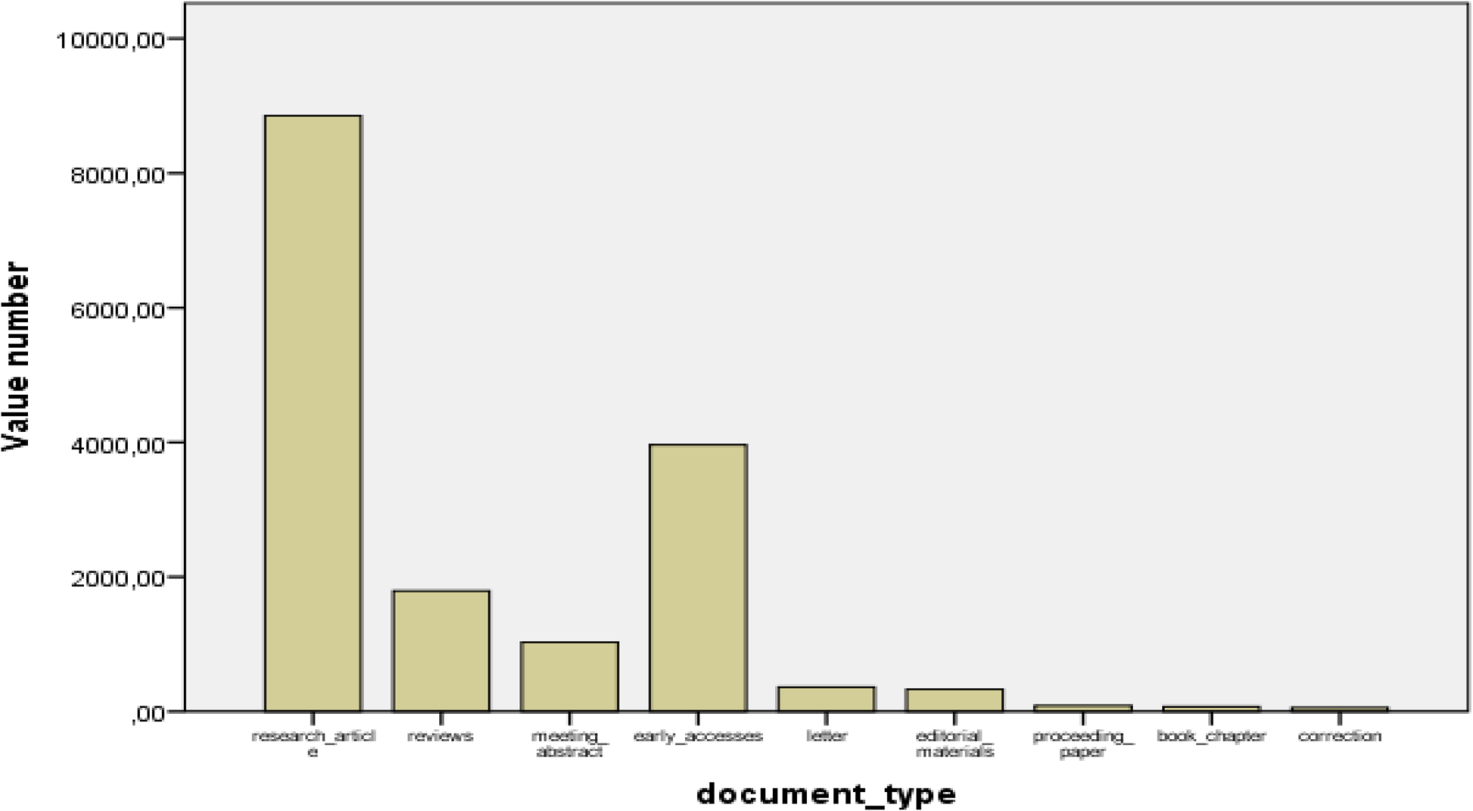
The distribution of document types

Among 12,461 articles, according to document types, 8855 were research articles (71.0%, highest), 1793 were reviews (14.3%), 1028 meeting abstracts (8.2%), 3963 were early accesses (3.1%), 364 were letters (2.9%), 330 were editorial materials (2.6%), and others (proceeding paper, correction, book chapter, etc.) (Fig. 1). A total of 7446 were open accesses.

**Fig. 2:**
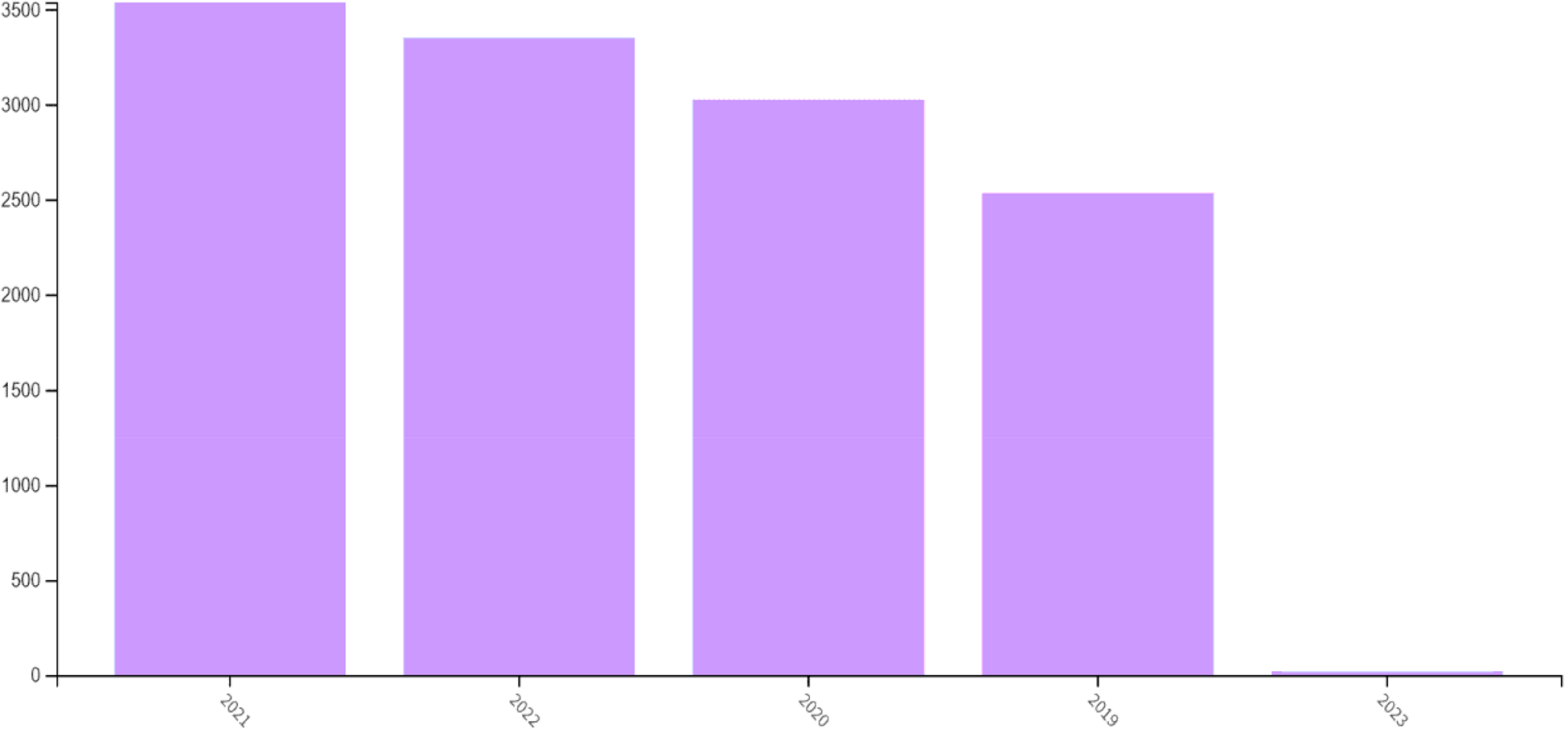
The bar graphic of the number of papers according to publication year (2019–2022)

The number of papers according to publication year was as shown at the bar graphic (Fig. 2). A total of 3537 were in 2021 (highest), 3349 were in 2022, 3024 were in 2020, 2532 were in 2019, and 19 were in 2023.

**Fig. 3:**
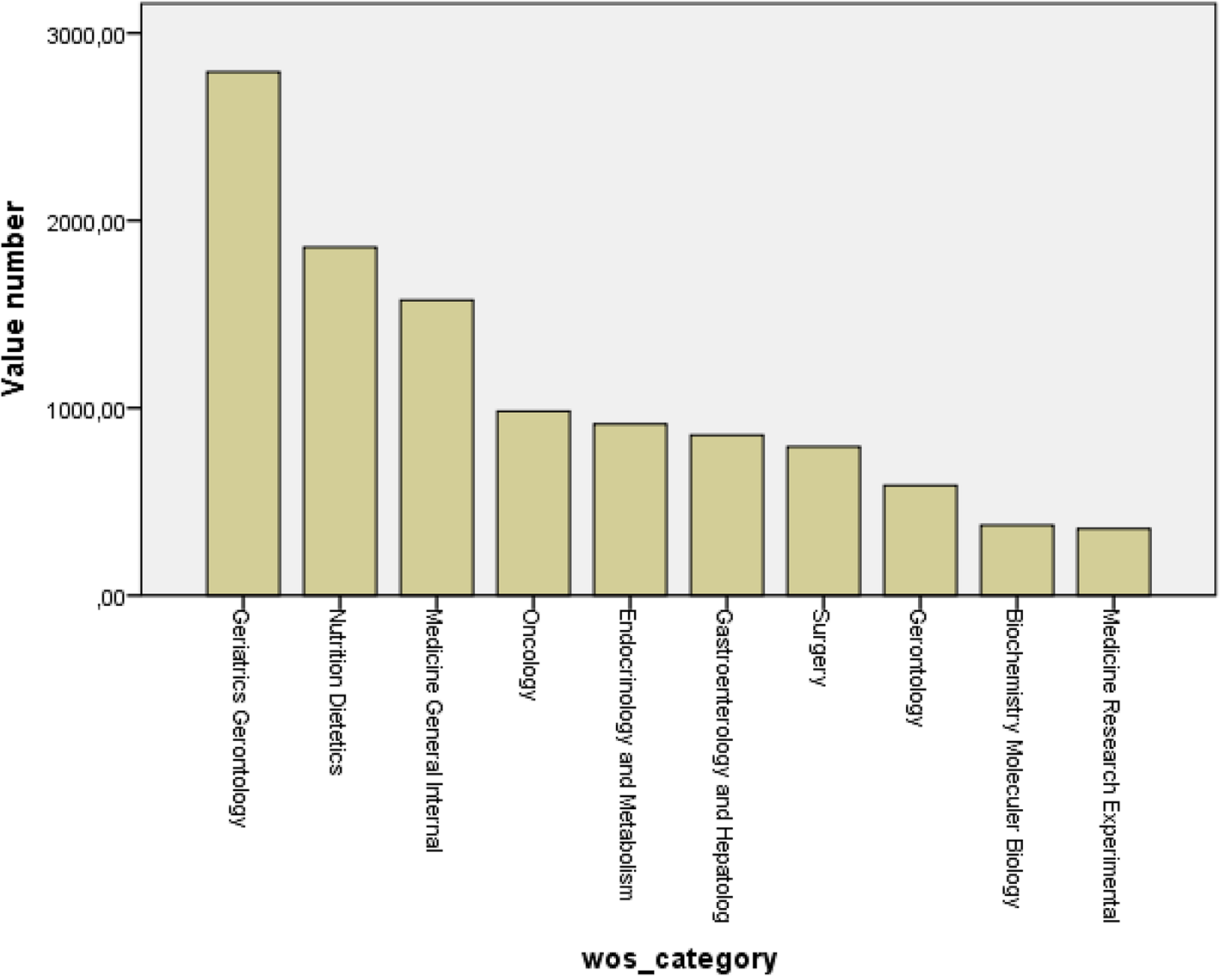
The graphic of the Web of Science categories list

According to the top 5 Web of Science categories list, 2792 were in Geriatrics Gerontology (highest), 1857 were in Nutrition Dietetics, 1575 were in Medicine General Internal, 983 were in Oncology, and 914 were in Endocrinology and Metabolism. Additionally, 243 paper were in Sport Sciences, 201 were in Rehabilitation, 196 were in Rheumatology, and 187 were in Clinical Neurology (Fig. 3 and Table 1).

**Table 1:** The distribution of top 10 WoS categories

Web of Science categories	Total (*N* = 9513)	Frequency (%)
Geriatrics Gerontology	2792	22.4
Nutrition Dietetics	1857	14.8
Medicine General Internal	1570	12.6
Oncology	983	7.8
Endocrinology and Metabolism	914	7.3
Gastroenterology and Hepatology	855	6.8
Surgery	794	6.3
Gerontology	587	4.7
Biochemistry, Molecular Biology	375	3.0
Medicine Research, Experimental	356	2.8

**Fig. 4:**
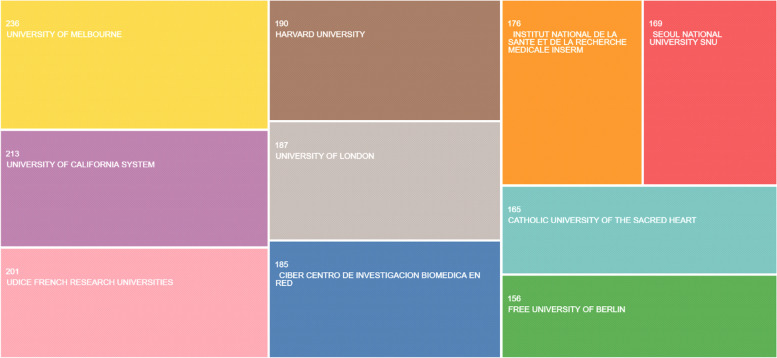
The distribution of top affiliations

**Fig. 5:**
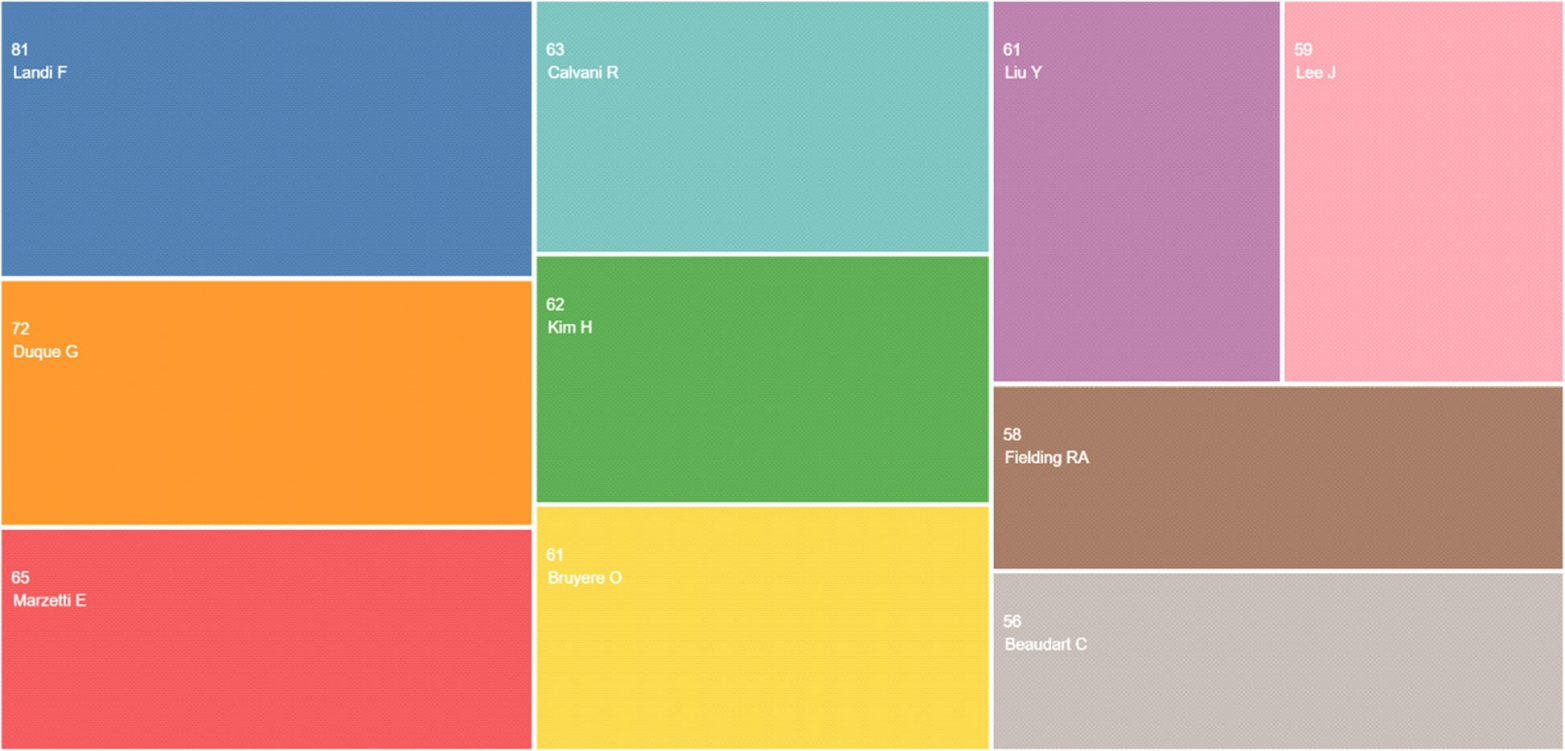
The distribution of top researchers

The distribution of top 10 affiliation is shown in Table 2 and Fig. 4; the distribution of top researchers is shown in Table 3 and Fig. 5.

**Table 2:** The distribution of top 10 affiliations

**Affiliations**	**Total (*N* = 1878)**	**%**
University of Melbourne	236	1.8
University of California System	213	1.7
UDICE French Research Universities	201	1.6
Harvard University	190	1.5
University of London	187	1.5
Ciber Centro Di Investigacion Biomedica En Red	185	1.4
Institut National De La Sante Et De La Recherche Medicale Inserm	176	1.4
Seoul National University (SNU)	169	1.3
Catholic University of Sacred Heart	165	1.3
Free University of Berlin	156	1.2

**Table 3:** The top 5 researchers in the WoS

Researchers	Number of paper	Affiliation	Country
Landi, Francesco	80	Catholic University of Sacred Heart	Italy
Duque, Gustavo	65	McGill University	Canada
Marzetti, Emanuele	63	IRCCS Policlinico Gemelli	Italy
Byuyere, Olivier	58	University of Liege	Belgium
Reginsten, Jean Yves	55	King Saud University	Saudi Arabia

According to the countries and areas, the top list was as follows: 1st the USA (2192), 2nd Japan (1924), and 3rd People’s Republic of China (1028). The most frequently used language is English with a number of 12,242.

**Fig. 6:**
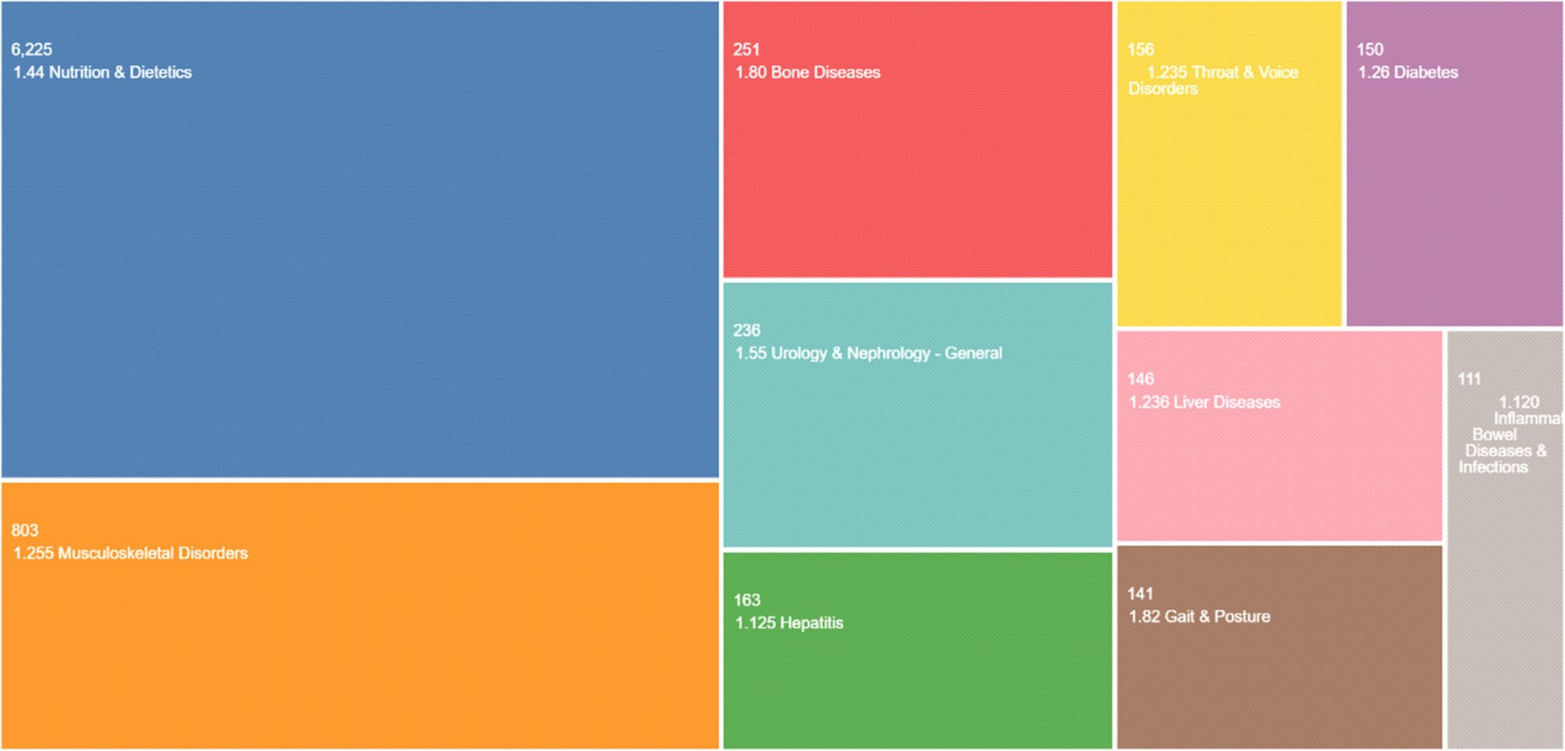
The graphic of the top citation topics

Top citation topics were Nutrition and Dietetics (6225), Musculoskeletal Disorders (803), Bone Disease (251), Urology & Nephrology-General (236), and Hepatitis (163), respectively (Fig. 6).

The distribution of the top 5 publishers is listed as follows: 1st Springer Nature (2295), 2nd Elsevier (2200), 3rd Wiley (2003), 4th MDPI (1423), and 5th Frontiers Media SA (544), respectively. When we add the key word “fragility” in the second row, the number of papers is 105.

**Fig. 7:**
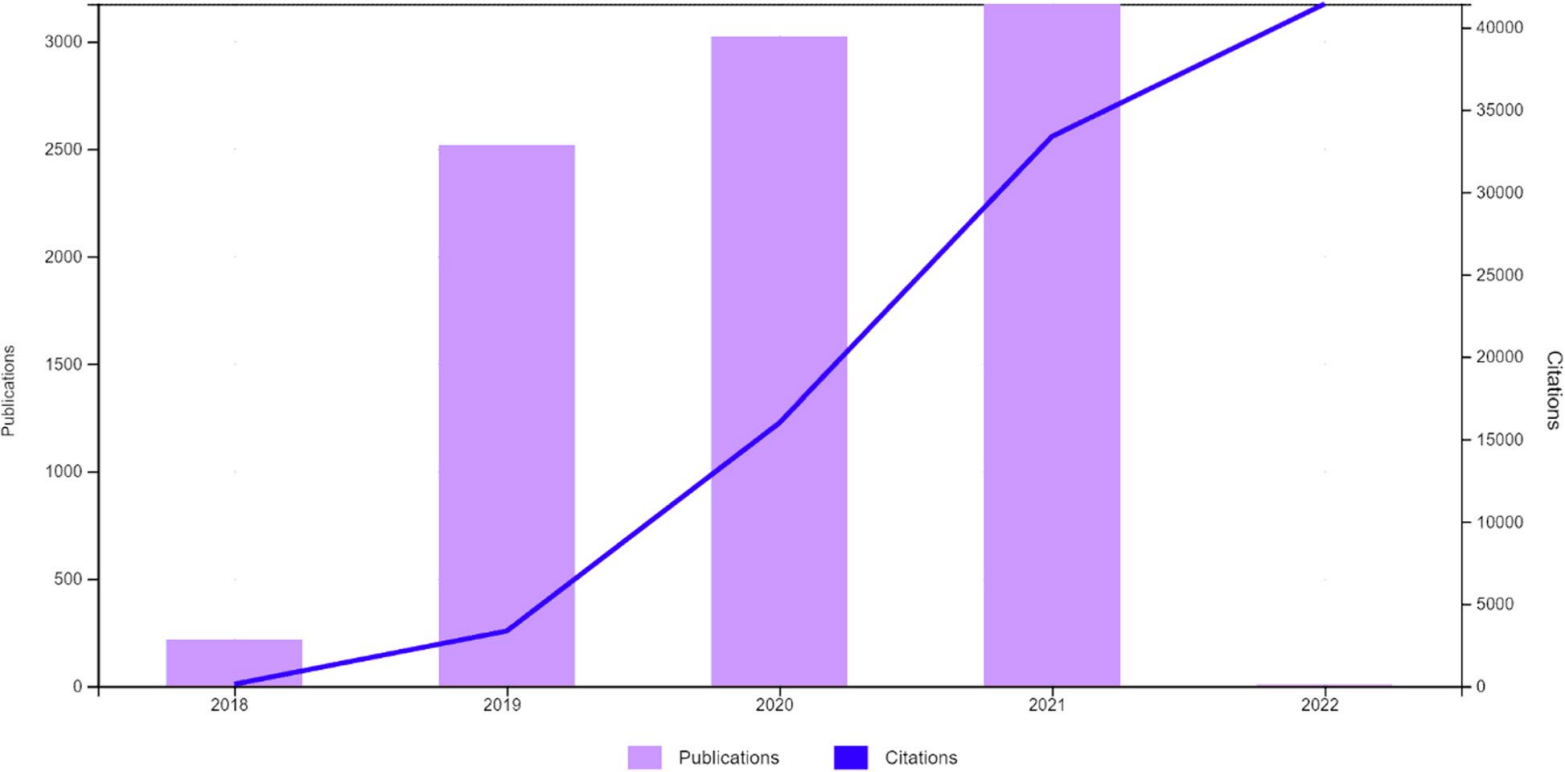
The bar graphic of the citing articles between 2018 and 2022

The number of citing articles, between 2018 and 2022 years, was 39,534 (with self-citations) and 39,088 (without self-citations); times cited was 94,584 (with self-citations) and 69,585 (without self-citations) as shown in Fig. 7.

The distribution of the top 10 documents in terms of citations is shown in Table 4.

**Table 4:** The distribution of the top 10 documents in terms of citations between 2018 and 2022

Document title	Authors	Years	Source	Number of citation
Sarcopenia: revised European consensus on definition and diagnosis	Cruz-Jentoft, Alfonso J. et al	2019	Age and aging	3821
Asian Working Group for Sarcopenia: 2019 consensus update on sarcopenia diagnosis and treatment	Chen, Liang-Kung et al	2020	*Journal of The American Medical Directors Association*	1335
Sarcopenia	Cruz-Jentoft et al	2019	*Lancet*	815
GLIM criteria for the diagnosis of malnutrition: a consensus report from the Global Clinical Nutrition Community	Cederholm, T. et al	2019	*Clinical Nutrition*	766
GLIM criteria for the diagnosis of malnutrition: a consensus report from the Global Clinical Nutrition Community	Jensen, Gordon L. et al	2019	*Journal of Parenteral and Enteral Nutrition*	766
GLIM criteria for the diagnosis of malnutrition: a consensus report from the global clinical nutrition community	Cederholm, T. et al	2019	*Journal of Cachexia Sarcopenia and Muscle*	443
Sarcopenia: aging-related loss of muscle mass and function	Larsson, Lars et al	2019	*Physiological Reviews*	419
Resistance training for older adults: position statement from the National Strength and Conditioning Association	Fragala, Maren S. et al	2019	*Journal of Strength and Conditioning Research*	343
Effects of time-restricted eating on weight loss and other metabolic parameters in women and men with overweight and obesity: the TREAT Randomized Clinical Trial	Lowe, Dylan A. et al	2020	*JAMA Internal Medicine*	337
Ethical guidelines for publishing in the *Journal of Cachexia, Sarcopenia and Muscle*: update 2019	von Haehling, Stephan et al	2019	*Journal of Cachexia Sarcopenia and Muscle*	305

**Fig. 8:**
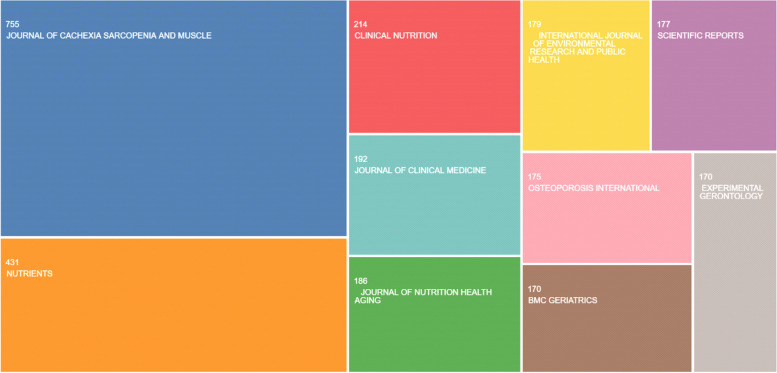
The distribution of publication titles

The top publication titles are listed as follows: *Journal of Cachexia*, *Sarcopenia and Muscle* (*N* = 755, 6.0%), *Nutrients* (*N* = 431, 3.4%), *Clinical Nutrition* (*N* = 214, 1.7%), *Journal of Clinical Medicine* (*n* = 192, 1.5%), and *Journal of Nutrition Health Aging* (*N* = 186, 1.4%), respectively (Fig. 8).

The indexes of the papers are listed as follows: the number of Science Citation Index-Expanded (SCI-E) was 11,324 (90.8%), Social Science Citation Index (SSCI) was 2573 (20.6%), and Emerging Sources Citation Index (ESCI) was 1007 (8.0%).

## Discussion

When we look at the articles in the fields of sarcopenia, we see more articles in the fields of Geriatrics and Nutrition. With the covid-19 pandemic that shook the world in 2019, both access to health services became difficult, and physical activity was restricted for all individuals. In addition, patients in the intensive care unit faced both immobilization and malnutrition. We encountered sarcopenia in the last decade, which often accompanies immobilization, lack of physical activity, and malnutrition, more frequently than before. 2021 has been the year with the most articles in sarcopenia.

When trying to cope with the primary disease, clinicians generally do not take into account the accompanying factors such as sarcopenia, which negatively affects both the treatment and quality of life of the individual. For these reasons, research on sarcopenia remains limited, and the diagnosis and treatment guidelines for sarcopenia cannot be developed adequately. Primary healthcare professionals should identify risk factors for age-related and disease-related sarcopenia, especially risk factors such as low physical activity and sedentary life. More clinical studies are needed to measure muscle mass with appropriate methods. Sarcopenia is a separate concept from myopenia, which is disease-related muscle mass [8]. Sarcopenia should be considered in routine examinations in people with advanced age and chronic diseases.

The increase in life expectancy worldwide results in an increase in the number of the elderly population. Sarcopenia decreases the quality of life in relation to the decrease in self-care [9, 10]. Sarcopenia is an important component of frailty syndrome and is a cause of disability, morbidity, and mortality in elderly individuals [11, 12]. Impaired mobility may result in an increased risk of falls and fractures, impaired daily activity performance, disability, loss of independence, and increased risk of death. Geriatrics and Gerontology rank first in the WoS categories related to sarcopenia. From this point of view, it necessitates that sarcopenia be included in the components of a public health issue [13]. Sarcopenia is frequently observed in chronic disease-related nutritional disorders. Nutrition Dietetics is in the 2nd place in WoS categories.

Sarcopenia may also develop in young individuals with autoimmune diseases [4, 14]. Sarcopenia often develops due to multifactorial metabolic causes (chronic inflammation, oxidative stress, protein degradation, reduced muscle blood flow, adipose tissue infiltration, etc.) in individuals with chronic rheumatic disease and negatively affects the physical functions and quality of life [15, 16]. Despite this, we found relatively few documents, *N* = 196, in the field of “Rheumatology” compared to other categories. The development of scientific studies in the field of geriatrics has led to increased awareness of sarcopenia and the necessity of an approach that concerns different disciplines. In our analysis, Geriatrics, Nutrition, Internal Medicine, and Oncology rank first among many different scientific disciplines.

Considering sarcopenia at the beginning of the treatment, management and rehabilitation will increase effectiveness of the treatment and the well-being. We see that the subject of sarcopenia is not sufficiently interested in the rehabilitation field (with a number of 201 papers). In addition to sarcopenia, fragility should be evaluated together. The deficiency of publications in this area is due to the inadequacy of standardized criteria for the diagnosis of sarcopenia and not paying enough attention. Insufficient understanding of the multifactorial and complex pathogenesis of the sarcopenia reduces the success of both diagnosis and treatment. The evaluation of sarcopenia and its subject to research should be increased in the fields of both rehabilitation and rheumatology.

Bibliometric analyses are used to investigate the impact of publications in any scientific field worldwide. Bibliometric analysis reviews published literature using quantitative and qualitative data. In this way, productivity in the academic field can be analyzed objectively. By the analysis of citations, the influence of articles on each other is also revealed.

The primary purpose is to scan databases in top trending areas with a comprehensive perspective [17, 18]. The USA is the country that carries out the most scientific studies in the field of sarcopenia. Japan takes second place. The high number of sarcopenia researches in these countries may be due to the fact that sarcopenia is among the preliminary evaluations in their healthcare services or due to the large proportion of elderly population. It may also be due to differences in cancer prevalences among countries. We can say that sarcopenia has received more attention in healthcare services in these countries and has led to more scientific articles.

When we look at the top authors, individually, in the field of sarcopenia, the first author is from Italy, and the second is from Canada. We see that the most cited articles are about diagnostic criteria of sarcopenia and from the Asian and European Study Groups. The increasing number of citations over the past years, 2019–2021, supports the increasing scientific interest in the field of sarcopenia. The 2019 covid pandemic has caused an increase in sarcopenia prevalence for many reasons such as immobilization, malnutrition, and stay in intensive care unit.

## Conclusion

We see that sarcopenia has been the subject and focus of researches in various disciplines in the last 5 years. The prevalence of sarcopenia among autoimmune diseases varies, and sarcopenia contributes to morbidity and mortality to these patients. We hope to increase the awareness of sarcopenia in the different scientific areas dealing with the chronic rheumatic diseases, disability, and geriatrics, and it takes its place in our routine clinical examinations in the future.

## Data Availability

If necessary, the data will be given by the author.

## Abbreviations

WoS: Web of Science
USA: United States of America
ESCI: Emerging Sources Citation Index
SCI-E: Science Citation Index-Expanded
SSCI: Social Science Citation Index
